# Supplementary material for: Complex‐centric proteome profiling by SEC‐SWATH‐MS
Source: Mol Syst Biol. 2019 Jan 14;15(1):e8438. doi: 10.15252/msb.20188438 (PMC6346213; doi:10.15252/msb.20188438)

Q68CZ6_Q96CS2_O94927_Q9H6D7
Annotated subunits: 7   Subunits with signal: 7
Max. coeluting subunits: 3   Max. completeness: 0.43

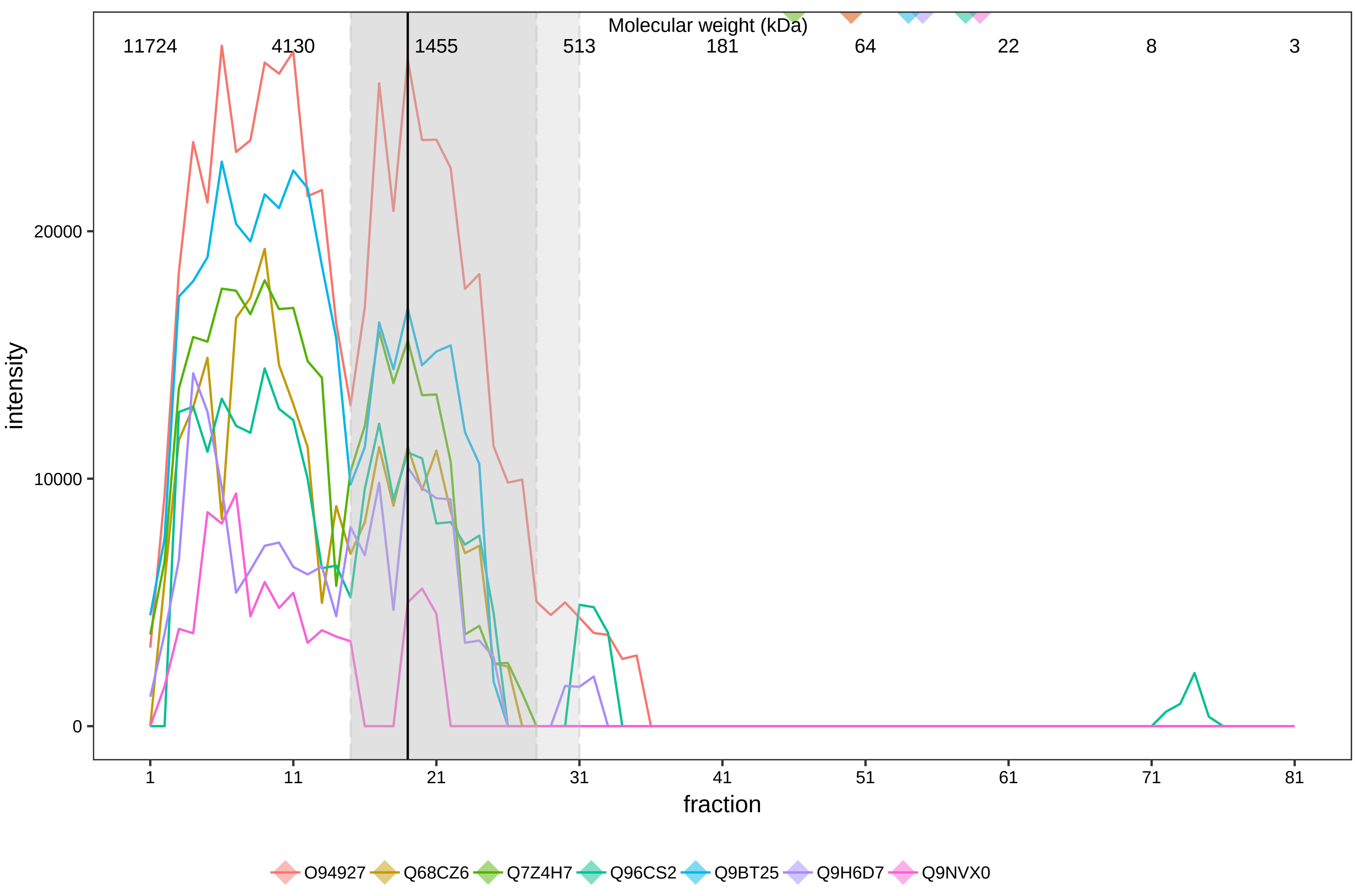

Supplement: Supplementary file 8 — Dataset EV7 [file MSB-15-e8438-s008.zip › feature_plots_string/Q68CZ6.pdf]
